# Factors Associated With Health-Related Quality of Life among Hypertensive Patients in Kathmandu, Nepal

**DOI:** 10.3389/fcvm.2017.00069

**Published:** 2017-11-06

**Authors:** Saruna Ghimire, Praful Pradhananga, Binaya Kumar Baral, Naveen Shrestha

**Affiliations:** ^1^Agrata Health Education and Development (AHEAD)-Nepal, Kathmandu, Nepal; ^2^Department of Biochemistry, Nepal Medical College and Teaching Hospital, Kathmandu, Nepal; ^3^School of Health and Allied Sciences, Pokhara University, Pokhara, Nepal

**Keywords:** quality of life, health-related quality of life, EuroQol-5Dimension, EuroQol visual analytic scale, hypertension, Nepal

## Abstract

**Introduction:**

Nepal has a high prevalence of hypertension. While improving the overall health-related quality of life (HRQOL) is a central tenet to public health plans in developed nations, this focus has yet to be articulated in Nepal. Therefore, this study aims to identify the factors associated with HRQOL among hypertensive patients in Nepal.

**Method:**

The EuroQol-5Dimension HRQOL survey was administered to 180 hypertensive patients, attending the outpatient clinic at Shahid Gangalal National Heart Center in Kathmandu, Nepal. Multiple linear regression models, adjusted for age and sex, were used to identify factors associated with HRQOL.

**Results:**

The mean age and EuroQol visual analytic scale of the participants were 53.2 years and 63.7, respectively. Age [β = −0.56; 95% confidence interval (CI): −0.75 to −0.37], income (β = 0.02; 95% CI: 0.01, 0.03), family size (β = −0.98; 95% CI: −1.89, −0.07), number of antihypertensive drugs use (β = 4.62; 95% CI: 1.33, 7.90), and compliance to dietary salt advise (β = 4.86; 95% CI: 0.29, 9.43) were significant factors associated with HRQOL among participants. In addition, levels of education and self-perceived health were associated in a positive gradient to HRQOL. In mediation analysis, both, dietary low salt compliance and use of antihypertensive drugs, had a significant direct effect on HRQOL, and the use of antihypertensive drugs did not significantly mediate the relationship between dietary salt compliance and HRQOL.

**Conclusion:**

Various factors were found to be associated with HRQOL among hypertensive patients in Kathmandu. Assessing HRQOL is a valuable technique to identify populations in need of services and interventions. This assessment can serve as a baseline, and in conjunction with multiple stakeholders, can guide public health policy, planning, and practices, especially those aimed toward improving the HRQOL of Nepalese with hypertension.

## Introduction

Hypertension is a global public health issue and a leading cause of cardiovascular disease (1), accounting for 45% of deaths due to heart disease, and 51% of deaths due to stroke globally (2). Nepal, a country in epidemiological transition, is facing alarmingly increasing prevalence of hypertension (3). Studies conducted in various parts of Nepal showed hypertension prevalence ranging from 9.9 to 41.8% (3–5). A repeated survey in a village of Nepal showed a threefold increment in the prevalence of hypertension in the same location after 25 years (3).

The health-related quality of life (HRQOL) assessments provide critical subjective information on an individual’s perception of mental and physical health as experienced in daily life (6). Biomedical measures of population-based health, including mortality and morbidity rates, provide only a partial picture of the general health and well-being of a population (7). While improving the overall HRQOL is a central tenet to public health plans in developed nations (8), and HRQOL surveillance is used to identify subgroups with relatively poor perceived health and guide the interventions (7), this focus has yet to be articulated in Nepal. Many international studies have found that hypertension interferes with the HRQOL of hypertensive patients (9–11). HRQOL of hypertensive patients is lower compared with normotensive people (12). Similar studies in context to Nepal are lacking. Owing to the sociodemographic and cultural discrepancies between Nepal and these countries, an inference cannot be made on HRQOL of Nepalese hypertensive patients from these international studies. A recent survey done among hypertensive patients in Manmohan Cardiothoracic Vascular and Transplant Centre in Kathmandu Nepal found age, non-formal education, and being single to be associated with lower HRQOL (13). For the first time in 2009, Nepal included non-communicable diseases (NCDs) in national health policy (14). However, implementing such policies into practice will require knowledge on various aspects of NCDs, including hypertension. Therefore, this study aims to identify the factors associated with HRQOL among hypertensive patients in Kathmandu, Nepal.

Furthermore, both dietary salt reduction and antihypertensive drugs use contributes significantly to increase the HRQOL among hypertensive patients (13, 15). According to dietary approaches to stop hypertension dietary salt reduction is one of the most cost-effective and most easily implemented strategies in controlling hypertension (16). The trials of hypertension prevention reported significant improvement in the psychological construct of HRQOL scale among low sodium group participants (15). Therefore, we hypothesize that dietary salt compliance is positively associated with participants HRQOL. However, it is not clear if dietary salt restriction independently impacts the HRQOL or whether this relationship is mediated by the use of antihypertensive drugs and thus requires further evaluation. Therefore, a secondary aim of our study is to assess the mediating role of antihypertensive drug used in the hypothesized relationship between dietary salt compliance and HRQOL.

## Materials and Methods

### Study Setting

This study was conducted on June–July 2015, in the outpatient clinic at Shahid Gangalal National Heart Center (SGNHC) in Kathmandu, Nepal. SGNHC is the national referral center for cardiology and cardiac surgery in Nepal.

### Study Population

To be eligible, the participants should be (1) a hypertensive patient, (2) at least 18 years, (3) be on a follow-up visit on the day of data collection, and (4) must have Out Patient Department (OPD) card that reflected their medical history. Hypertensive patients were defined by systolic blood pressure above 140 mmHg or diastolic blood pressure above 90 mmHg. Eligibility criteria for participants were ascertained through their OPD card. Patients admitted to inpatient clinic and those with severe cardiac disease, and surgery was excluded. Surveyors, supervised by the first author, were graduates of public health with prior experience of survey data collection. The surveyors were provided with an orientation on the EuroQol-5Dimension (EQ-5D) tool, sampling strategy, and data collection techniques prior to data collection. At SGNHC, almost all patients attend OPD without a prior appointment. Thus, due to the absence of a sampling frame, all eligible participants were recruited until the desired sample size was achieved.

### Sample Size

The sample size of 180 was calculated with reference to Kittle (17), with a *p*-value of 0.05, an alpha error of 5%, and a power of 80%.

### Data Collection and Variables

#### EuroQol Five-Dimension Three-Level (EQ-5D-3L) Variables

Individual interview was conducted during patients waiting time using Nepali versions of European quality of life tool (EQ-5D), described elsewhere (18). The reliability and validity of Nepali versions of EQ-5D were established in a previous study (19). The EQ-5D-3L allows participants to classify their health status in five different dimensions (i.e., mobility, self-care, usual activities, pain/discomfort, and anxiety/depression) and within three-level response (no problems, moderate problems, and severe problems) (20). The five dimension and three-level response of EQ-5D represent 243 (3^5^) various health states (20).

#### EQ-5D Index

From the five dimensions of ED-5D, a single index value, called EQ-5D index was calculated in “EQ-5D-3L Crosswalk Index Value Calculator” (21), using weights of UK as the reference. The EQ-5D index ranges from 0 to 1, where 0 indicates severely ill, and 1 indicates a perfect health. Perfect health is represented by no problems on all five dimensions (11111) and is assigned an index value of 1. Likewise, very severe health states, corresponding to severe problems on all of the five dimensions (33333), received 0 values.

#### EuroQol Visual Analytic Scale (EQVAS)

EuroQol visual analytic scale is a vertically calibrated scale that allows participants to rate their overall health on a scale ranging from 0 to 100; where 0 and 100 signify the worst and the best imaginable health state, respectively. During data collection, each participant was given a pen and asked to indicate the point on EQVAS that they felt best described their overall health on that day.

### Sociodemographic Variables

Sociodemographic variables included age, gender, ethnicity, marital status, educational status, occupation, monthly income of the family, family size and type, self-perceived health and compliance to low salt diet. For ethnicity, the Nepal Health Management Information System’s “caste/ethnic groupings” were used (22). Marital status was categorized as married, unmarried and widow/separated. Education status was categorized into four groups as follows: illiterate; informal (no formal schooling and some literacy); formal education (any years of formal schooling); and a university education (education beyond high school). Nepal Standard Occupation Classification NSOC-99 (23) was used for the occupational category. Monthly family income of participants was self-reported in Nepalese rupees (NRs), which was converted into US dollar (1$ = 100 NRs) during data analysis. Family type was recorded as a nuclear, joint, and extended family. Antihypertensive drugs use was recorded from patients OPD card. A screening tool developed with reference to “the practical guidelines for conducting a barrier analysis” (17) was used to classify participants as compliant or non-compliant to low dietary salt.

### Data Processing and Statistical Analysis

Study tool was pretested among 18 hypertensive patients meeting the study inclusion criteria at Heart Clinic, Kathmandu. Pretest responses were not included in the final analysis. Data were managed in EpiData. Statistical analyses were performed in IBM SPSS22 for Windows (SPSS Inc., Chicago, IL, USA). Descriptive statistics (frequency, proportion, mean, and SE) were calculated for all variables. The Shapiro–Wilks test was used to check the normality of variable distributions. Frequency distributions were compared by Pearson’s chi-square (χ^2^) test, and means between the groups were compared by Student’s *t*-test or analysis of variance. Multiple linear regression models, adjusted for age and sex, were used to determine the factors associated with HRQOL. Pearson’s correlation coefficient was used to determine the relationship between the EQ-5D-3L index values and the EQVAS scores.

A mediational analysis (24) was conducted to determine if the use of antihypertensive drugs might influence the association between dietary salt compliance and HRQOL. Mediation analysis was conducted using PROCESS macro for SPSS (25) following non-parametric bootstrap approach (26). The macro tests mediation models by comparing the observed indirect effect against 5,000 bootstrapped resamples (24). The coefficients of effects and their bias accelerated and corrected (BCa) 95% confidence intervals were obtained from 5,000 random bootstrap samples. The bootstrapping method is more statistically robust than the Sobel test (24) and is highly recommended to examine indirect mediation effects (24). BCa confidence intervals that do not include a 0 indicate a statistically significant effect estimates (24). The mediation analysis was first ran without any covariates, then adjusted for age, sex, and monthly family income. For all statistical tests, two-tailed *p*-values < 0.05 were considered statistically significant.

## Results

### Demographic Characteristics of the Study Participants

Table 1 provides the descriptive characteristics of the study participants. One hundred eighty hypertensive patients, an equal number of males and females, participated in the study. The overall response rate for each EQ-5D question was 100%. The mean age (±SD) of the participants was 53.2 ± 12.6 years. A majority of the participants were married (91.1%), upper caste (41.7%), had formal education (55.0%), and lived in a nuclear family (55.6%). At the time of the study, more than half (51.7%) of the participants were involved in elementary occupations (low-skilled manual jobs) and had mean (±SD) family income (per month) of $269.19 ± 154.37 (Table 1).

**Table 1:** Descriptive characteristics of the study participants (*n* = 180).

Characteristics		EQVAS	*p*-Value
		Mean ± SE: 63.7 ± 1.3	
Age, mean ± SD	53.2 ± 12.6		
Gender, *n* (%)			0.540^a^
Female	90 (50.0)	63.2 ± 1.9	
Male	90 (50.0)	64.7 ± 1.7	
Ethnicity, *n* (%)			0.510^b^
Dalit	5 (2.8)	56.8 ± 7.3	
Disadvantaged Janajatis	48 (26.7)	63.4 ± 2.5	
Disadvantaged non-Dalit Terai caste	8 (4.4)	56.5 ± 6.8	
Relatively advantaged Janajatis	44 (24.4)	63.6 ± 2.6	
Upper caste groups	75 (41.7)	65.8 ± 1.9	
Marital status, *n* (%)			**0.008^a^**
Widow	16 (8.9)	53.1 ± 4.5	
Married	164 (91.1)	65.0 ± 1.3	
Educational status, *n* (%)			**<0.001^b^**
Illiterate	22 (12.2)	49.0 ± 3.6	
Informal	39 (21.7)	59.8 ± 2.7	
Formal	99 (55.0)	67.2 ± 1.6	
University education	20 (11.1)	72.4 ± 3.1	
Occupation, *n* (%)			0.052^b^
Elementary occupations	93 (51.7)	61.2 ± 1.9	
Clerical, service, and sales workers	32 (17.8)	66.8 ± 3.0	
Professionals	36 (20.0)	69.7 ± 2.5	
Agricultural workers	19 (10.6)	61.5 ± 3.0	
Monthly family income $, mean ± SD	296.1 ± 169.8		
Monthly family income $, *n* (%)			**0.026^b^**
<200	53 (29.4)	59.0 ± 2.4	
200–500	107 (59.4)	65.4 ± 1.6	
>500	20 (11.1)	69.5 ± 3.2	
Family size, mean ± SD	6.1 ± 2.7		
Family type, *n* (%)			0.053^a^
Nuclear	100 (55.6)	66.1 ± 1.7	
Joint	80 (44.4)	61.2 ± 1.8	
Salt compliance, *n* (%)			**0.023^a^**
Non-compliant	90 (50.0)	61.1 ± 1.7	
Compliant	90 (50.0)	66.8 ± 1.8	
Antihypertensive drugs use			**<0.001^b^**
0	27 (15.0)	50.1 ± 3.6	
1	92 (51.1)	65.7 ± 1.4	
2 or more	61 (33.9)	67.4 ± 2.4	
Compare to past year current health status			**<0.001^b^**
Better	69 (38.3)	75.0 ± 1.7	
Similar	88 (48.9)	59.4 ± 1.4	
Worse	23 (12.8)	48.3 ± 3.9	

*ANOVA, analysis of variance; EQVAS, EuroQol visual analytical scale; $, US dollar*.

*Mean difference between the groups by ^a^t-test and ^b^ANOVA*.

*Statistically significant values are bolded*

### EQ-5D Descriptive Health Profile of the Study Participants

The descriptive health profile of the participants, based on the five dimensions of EQ-5D, is given in Table S1 in Supplementary Material. Many hypertensive patients reported having problems with pain (38%), sadness (38%), routine work (26%), mobility (17%) and self-care (11%). The findings were consistent among males and females, and there were no statistically significant differences between the genders (Table S1 in Supplementary Material).

### The EQVAS and EQ-5D Index Value of Study Participants

The mean (±SE) EQVAS score and EQ-5D-3L index of the participants were 63.7 ± 1.3 and 0.87 ± 0.01, respectively. A statistically significant moderate positive correlation was found between EQVAS and EQ-5D-3L index value (*r* = 0.35; *p* < 0.001). The mean EQVAS significantly differed on marital status, educational level, compliance with dietary salt advice, antihypertensive drugs use, and self-perceived health status (Table 1). The participants reported a total of 25 different states of health. Forty-five percent of the participants had full health (11111) score, whereas none of the participants had the worst health states (33333).

### Factors Associated With HRQOL

In univariate analysis, age, marital status, educational status, family income, family type and size, compliance to dietary salt advice, and self-perceived health status were significant predictors of HRQOL (Table 2). Whereas, when adjusted for age and gender, marital status and family type were no longer significant. For every unit increase in the age (years), the EQVAS score decreased by 0.56 unit. Compared with illiterate, HRQOL was higher among those with a formal [β = 11.86; 95% confidence interval (CI): 2.18, 21.54] or a university education (β = 15.95; 95% CI: 3.92, 27.98). Similarly, EQVAS score was increased by 0.02 for every one-dollar increase in family’s monthly income. Compared with non-compliant, participants compliant to dietary salt had higher HRQOL (β = 4.86; 95% CI: 0.29, 9.43). Family size was inversely associated with HRQOL (β = −0.98; 95% CI: −1.89, −0.07), whereas, the number of antihypertension medicines use was positively associated (β = 4.62; 95% CI: 1.33, 7.90). Compared with participants who had better-perceived health status, HRQOL was lower among participants who perceived that their health status was similar (β = −12.35; 95% CI: −17.01, −7.69) or have worsened (β = −23.39; 95% CI: −30.17, −16.61) over the past year (Table 2).

**Table 2:** Factors associated with health-related quality of life of study participants.

	Univariate		Multivariate^a^	
	β	95% CI	β	95% CI
Age	**−0.56**	**−0.75, −0.37**	**−0.56**	**−0.75, −0.38**
**Gender (reference = female)**					
Male	1.97	−3.20, 7.14	3.68	−0.98, 8.33
**Ethnicity (reference = upper caste)**								
Dalit	−8.96	−24.99, 7.07	−9.08	−23.70, 5.55
Disadvantaged Janajatis	−3.16	−9.57, 3.26	−4.50	−10.37, 1.36
Disadvantaged non-Dalit Terai caste	−9.26	−22.17, 3.65	−7.19	−19.07, 4.69
Relatively advantaged Janajatis	−2.12	−8.72, 4.47	−1.52	−7.53, 4.50
**Marital status (reference = widow)**								
Married	**11.65**	**2.72, 20.58**	2.76	−6.26, 11.78
**Education (reference = illiterate)**								
Informal	**12.41**	**3.90, 20.92**	7.88	−1.17, 16.92
Formal	**19.76**	**12.24, 27.29**	**11.86**	**2.18, 21.54**
University education	**24.94**	**15.08, 34.80**	**15.95**	**3.92, 27.98**
Income, $	**0.02**	**0.01, 0.04**	**0.02**	**0.01, 0.03**
Family size	**−1.75**	**−2.66, −0.84**	**−0.98**	**−1.89, −0.07**
**Family type (reference = nuclear)**								
Joint	**−5.4**	**−10.55, −0.25**	−0.84	−5.86, 4.18
**Salt compliance (reference = non-compliant)**
Compliant	**5.77**	**0.80, 10.73**	**4.86**	**0.29, 9.43**
Antihypertension drugs use	**5.53**	**2.05, 9.01**	**4.62**	**1.33, 7.90**
**Self-perceived health status (reference = better)**
Similar	**−15.60**	**−20.14, −11.05**	**−12.35**	**−17.01, −7.69**
Worse	**−26.67**	**−33.47, −19.86**	**−23.39**	**−30.17, −16.61**

*^a^Adjusted for age and gender*.

β, unstandardized regression coefficient; CI, confidence interval.Statistically significant values are bolded

### Findings from Mediation Analysis

Table 3 presents the findings from mediational analysis with the antihypertensive drugs used as the mediator. Unadjusted mediation analysis showed that antihypertensive drugs use mediated 8% of the total effect of dietary salt compliance on HRQOL. However, the mediating effect of antihypertensive drugs use was not significant when adjusted for age, sex, and family income.

**Table 3:** Mediation analysis for the association between dietary salt compliance and health-related quality of life, mediated by antihypertensive medication use.

	Model 1		Model 2	
	β (SE)	BCa 95% CI	β	BCa 95% CI
Total effect, *c*	12.97 (2.36)	8.31, 17.64	11.31 (2.18)	7.01, 15.61
Direct effect, *c*′	12.00 (2.36)	7.34, 16.67	10.66 (2.19)	6.35, 14.98
Indirect effect, *ab*	0.97 (0.77)	0.02, 3.18	0.65 (0.58)	−0.03, 2.36
Ratio of indirect to total effect mediated	0.08		0.06	
Ratio of indirect to direct effect	0.08		0.06	

*Model 1: unadjusted mediational model*.

*Model 2: adjusted for age, sex, and monthly family income*.

*Number of bootstrap samples for bias-corrected bootstrap confidence intervals: 5,000*.

*β, unstandardized coefficient; BCa, bias accelerated and corrected*.

## Discussion

We aimed to assess the HRQOL of hypertensive patients in Kathmandu, Nepal and found that participants have self-perceptions of a low HRQOL in different dimensions of EQ-5D. The previous study evaluating HRQOL among hypertensive patient in Kathmandu (13) reported increasing age, not cohabiting with a partner, no formal education to be associated with lower HRQOL. We confirm their findings and additionally we also provide evidence that a higher HRQOL was associated with higher family income, participant’s dietary low salt compliance, use of antihypertensive drugs, and better-perceived health status. Mediation analysis conducted to further evaluate the association between antihypertensive drug use, dietary salt compliance, and HRQOL showed that both, dietary low salt compliance and use of antihypertensive drugs, had a significant direct effect on HRQOL. Further, the use of antihypertensive drugs did not significantly mediate the relationship between dietary salt compliance and HRQOL. This finding implies that both dietary salt compliance and antihypertensive drugs use are two independent aspects of HRQOL of hypertensive patients and both are independently essential to increase the HRQOL of hypertensive patients. Both, dietary salt compliance and antihypertensive drugs use help to control the blood pressure of hypertensive patients, and better-controlled blood pressure may lead to improved HRQOL. Previous research has demonstrated that non-adherence to prescription is associated with poor HRQOL (27, 28). Adherence to treatment leads to controlled blood pressure (29) and better health outcomes (30). Likewise, a modest reduction in dietary sodium intake is associated with significant reduction in blood pressure and a 20% reduction in cardiovascular events (31). A decrease in salt intake of 6 g/day lowered diastolic blood pressure by 7/4 mm of Hg in hypertensive patients and 4/2 mm of Hg in normotensive subjects (31). As reducing dietary salt has been considered as a non-pharmacotherapy for controlling blood pressure, the high HRQOL perceived by the hypertensive patient compliant to low dietary salt may be explained by the same mechanism that explained the difference in HRQOL between adherent and non-adherent patients.

A greater proportion of study participants reported having problems in the dimension of pain and sadness. Similar findings were reported by studies conducted to assess HRQOL among hypertensive patients using EQ-5D in other countries (9–11). A study evaluating HRQOL of the hypertensive patient in Kathmandu using 36-Item Short Form Survey questionnaire also found a higher mean score for mental health domain than the physical domain (13). As, headache and dizziness are the common symptoms of hypertension and hypertensive subjects have impaired psychological well-being (32), hypertension is thus likely to affect psychological dimension of HRQOL.

Among the study participants, HRQOL was significantly decreased by increasing age. Age is the strongest predictor of HRQOL (13, 33). During the life transition, various physical, mental, and psychosocial changes accompany the phenomenon of aging (34). Biologically, aging is characterized by a gradual, and lifelong accumulation of molecular and cellular damage that subsequently leads to a decrease in physiological functions increased vulnerability to diseases and a general decline in the capacity of the individual (35). As people age, there is a decline in vision and hearing (34), waned musculoskeletal strength and function (36), and compromised immune function (37). With increasing age, people are more likely to experience multimorbidity, which has a significant impact on HRQOL, the impact being greater than the additive effect of individual conditions (38).

In line with previous studies (10, 11), we found a significant positive association between HRQOL and educational level and income. Education is often used as a proxy for socioeconomic status and is highly associated with many health outcomes (39). Education is linked to both physical and mental health and has a direct positive relationship with HRQOL (40). The mechanisms underlying the relationship between health and education are equivocal (40). However, it is believed that education provides an individual with a wide range of utilitarian possessions that can be used for health advantage (40). Better educated are more likely to be better informed, have better critical thinking skills and decision-making abilities, adopt healthy lifestyle behaviors and preventive measures, and make use of health-related information to achieve better health outcomes and improved HRQOL (40).

Family income and economic status directly influence both physical and mental health status (41). The economic vulnerability impacts on health and HRQOL in terms of affordability of resources such as food, stable housing, transportation, and other daily needs (42). People with low income are more likely to be uninsured (43) and thus have limited access to health care. As incomes rise, willingness to pay for health improvements increases as well (44). Also, education and income is a way to achieve social status, social support and self-satisfaction, and identity (45). People with better education and better jobs have larger social networks that provide financial, physical, and emotional support; that may contribute to better health (46).

The number of family members was inversely associated with HRQOL. In a family with many members, the competing demands of many family members needs to be met, which may limit patients’ time and energy and introduce stress that can negatively affect the patients’ health outcomes (47).

In contrast to findings of studies from different countries (9, 11), this study and the previous study from Nepal (13) found no significant difference in HRQOL of hypertensive patients based on gender. The inconsistency may be explained by the fact that different society may have a different social position and opportunity between males and females. Considering the gender inequity in many aspects of Nepali society, including legal (48), socioeconomic (49), educational (50), and health (49, 51), a low HRQOL among Nepalese female, in general, is expected. However, based on this study and a previous study from Nepal (13), there appears to be no significant difference in HRQOL between hypertensive Nepalese male and female.

Health-related quality of life is higher among married people, compared with unmarried/separated (9, 13). Our univariate analysis showed that marital status was a statistically significant predictor of HRQOL. While the precise mechanisms by which marriage confers health benefits are unclear, studies show that married adults have better health and survival (52, 53). Married people have improved mental health compared with those who are single, divorced, or bereaved due to the social relationship with the spouse (54). Research also suggests that differences between the married and unmarried, including social, psychological, and financial resources, may all help to explain the mental health advantage of being married (55).

One of the limitations of this study is the relatively small sample size; nevertheless, the sample size falls within the recommended range for HRQOL studies using EQ-5D (56). An additional limitation of this study might be in the calculation of EQ-5D index value by using UK’s general population weight as the reference value. No such reference weights exist for Nepal and of the countries, for which the value sets are available (21), none of the countries closely resemble the socioeconomic context of Nepal. Therefore, the decision to use UK weights was made because EQ-5D was originally developed in the UK and a similar approach was also employed in a previous study conducted in Nepal, among pulmonary disease patients (19). Nevertheless, considering the huge socioeconomic difference between Nepal and UK such value may not be representative of the Nepalese health status. SGNHC has a diverse patient from different parts of the country. In this study too, more than 60% of the participants lived outside of Kathmandu Valley and were from 40 of the 75 districts in Nepal; therefore, may represent the diagnosed hypertensive patients in Nepal.

## Conclusion

This study evaluated the HRQOL among hypertensive patients attending OPD of SGNHC and identified age, educational status, family income and size, compliance with dietary salt advice, antihypertensive drug use, and self-perceived health status as the significant factors associated with HRQOL. Information on HRQOL is a valid indicator of service need and intervention outcomes (7) and is used to identify subgroups with relatively poor perceived health (7). The findings may be helpful for multiple stakeholders involved in health promotion of hypertensive patients in Nepal; to appraise public health policy, planning, and practice (7).

The traditional measure of disease morbidity and mortality provides an incomplete picture of population’s well-being (7). HRQOL combines both physical and mental aspects of health and thus gives a better picture of well-being. Although information on HRQOL forms the core tenant of public health programs in developed countries (8), such practice is lacking in Nepal. As Nepal is working toward implementing the recently formulated national policy on NCDs (14), therefore, the knowledge gained in this study may be helpful to understand the issue in local context better.

## Ethics Statement

Ethical approval for the study was provided by Ethical Review Board at Nepal Health Research Council, Kathmandu. Written approval was also taken from SGNHC. Each participant participated voluntarily and was given a detailed explanation of the purpose of the study by the surveyors; subsequently, written consent was taken from each participant before data collection. The identity of participants was kept confidential.

## Author Contributions

SG and NS conceptualized the study and developed methodology; developed the statistical analysis approach. SG analyzed the data. PP and BB facilitated data collection. SG, PP, and BB drafted the manuscript. All the authors critically reviewed and approved the final manuscript.

## Conflict of Interest Statement

The authors declare that the research was conducted in the absence of any commercial or financial relationships that could be construed as a potential conflict of interest.

## Abbreviations

ANOVA, analysis of variance; CI, confidence interval; EQ-5D, EuroQol-5Dimension; EQVAS, EuroQol visual analytic scale; HRQOL, health-related quality of life; NCDs, non-communicable diseases; NRs, Nepalese rupees; OPD, Out Patient Department; SF-36, 36-Item Short Form Survey; SGNHC, Shahid Gangalal National Heart Center.
