# Supplementary material for: Factors Associated With Health-Related Quality of Life among Hypertensive Patients in Kathmandu, Nepal
Source: Front Cardiovasc Med. 2017 Nov 6;4:69. doi: 10.3389/fcvm.2017.00069 (PMC5681715; doi:10.3389/fcvm.2017.00069)
Supplement: Supplementary file 1 [file Table_1.DOCX]

**Supplementary Table 1. EQ-5D descriptive health profile of the study participants (n=180)**

| **EQ-5D Dimensions** | **Total** | **Male** | **Female** | **p-value ^a^** |
| --- | --- | --- | --- | --- |
|  | **n (%)** | **n (%)** | **n (%)** |  |
| **Mobility** | | | | **0** **.167** |
| No problems | 149 (82.8) | 74 (82.2) | 75 (83.3) |  |
| Some problems | 28 (15.6) | 16 (17.8) | 12 (13.3) |  |
| Confined to bed | 3 (1.7) | - | 3 (3.3) |  |
| **Self-care** | | | | **0** **.239** |
| No problems | 160 (88.9) | 82 (91.1) | 78 (86.7) |  |
| Some problems | 20 (11.1) | 8 (8.9) | 12 (13.3) |  |
| **Routine work** | | | | **0** **.232** |
| No problems | 134 (74.4) | 72 (80.0) | 62 (68.9) |  |
| Some problems | 41 (22.8) | 16 (17.8) | 25 (27.8) |  |
| Unable to perform usual activity | 5 (2.8) | 2 (2.2) | 3 (3.3) |  |
| **Pain** | | | | **0** **.499** |
| No pain or discomfort | 112 (62.2) | 54 (60.0) | 58 (64.4) |  |
| Moderate pain or discomfort | 62 (34.4) | 34 (37.8) | 28 (31.1) |  |
| Extreme pain or discomfort | 6 (3.3) | 2 (2.2) | 4 (4.4) |  |
| **Sadness** | | | | **0** **.197** |
| Not anxious or depressed | 111 (61.7) | 59 (65.6) | 52 (57.8) |  |
| Moderately anxious or depressed | 63 (35.0) | 30 (33.3) | 33 (36.7) |  |
| Extremely anxious or depressed | 6 (3.3) | 1 (1.1) | 5 (5.6) |  |
| Abbreviations: EQ-5D, EuroQol 5-dimension  ^a^ Chi square test for differences by gender | | | | |
